# Protective effects of *Bacopa monnieri* against cisplatin-induced hepatorenal toxicity in rats: biochemical, oxidative stress, histopathological, and Kidney Injury Molecule-1–based evidence

**DOI:** 10.14202/vetworld.2026.1069-1084

**Published:** 2026-03-17

**Authors:** S. Sahana, Shivaprakash Gangachannaiah, Ravindra Maradi, K. G. Mohandas Rao, Sangita G. Kamath, Shalini Adiga, S. Chandana, Monalisa Biswas

**Affiliations:** 1 Department of Pharmacology, Kasturba Medical College, Manipal Academy of Higher Education, Manipal, Karnataka, India; 2 Department of Biochemistry, Kasturba Medical College, Manipal Academy of Higher Education, Manipal, Karnataka, India; 3 Department of Basic Medical Sciences, Manipal Academy of Higher Education, Manipal, Karnataka, India

**Keywords:** *Bacopa monnieri*, cisplatin hepatotoxicity, cisplatin nephrotoxicity, cisplatin-induced toxicity, hepatorenal protection, KIM-1 biomarker, oxidative stress, renal injury marker

## Abstract

**Background and Aim:**

Cisplatin, a platinum-based chemotherapeutic agent, is widely employed in the treatment of various malignancies. However, its clinical utility is restricted by dose-limiting hepatorenal toxicities, primarily driven by oxidative stress, inflammation, and direct cytotoxicity. The present *in vivo* study was designed to evaluate the protective efficacy of *Bacopa monnieri* extract against cisplatin-induced hepatorenal toxicity in male Wistar rats, utilizing biochemical parameters, oxidative stress biomarkers, histopathological assessment, and the early renal injury marker Kidney Injury Molecule-1 (*KIM-1*).

**Materials and Methods:**

Thirty male Wistar rats were randomly divided into five groups (n = 6 per group): control (normal saline), cisplatin (7.5 mg/kg intraperitoneally on day 7), and three intervention groups receiving *B. monnieri* (BM) extract orally at 100, 200, or 300 mg/kg daily for 10 consecutive days concomitantly with cisplatin. Urine samples were collected on day 9 for *KIM-1* quantification. On day 11, animals were euthanized, and blood, liver, and kidney tissues were obtained for biochemical assays (liver and kidney function tests, malondialdehyde, glutathione, superoxide dismutase, catalase) and histopathological evaluation.

**Results:**

Administration of *BM* at 200 and 300 mg/kg significantly attenuated cisplatin-induced elevations in serum alkaline phosphatase, aspartate aminotransferase, and alanine aminotransferase (all p < 0.001 versus the cisplatin group). Total protein and albumin levels were restored toward control values in the 300 mg/kg group. Urea concentrations decreased significantly at 300 mg/kg, while creatinine levels were reduced at both 200 and 300 mg/kg doses (p < 0.001). Urinary *KIM-1* concentrations remained elevated in all cisplatin-exposed groups without significant mitigation by *BM*. Dose-dependent improvements in antioxidant status were observed, with increased glutathione, superoxide dismutase, and catalase activities and decreased malondialdehyde levels in both liver and kidney tissues (p < 0.001 at higher doses). These biochemical findings were strongly supported by histopathological evidence showing reduced inflammatory cell infiltration, tubular necrosis, hydropic degeneration, sinusoidal dilatation, and fatty change, with near-normal tissue architecture restored at the 300 mg/kg dose.

**Conclusion:**

*BM* extract confers significant, dose-dependent hepatorenal protection against cisplatin-induced toxicity, predominantly through its potent antioxidant mechanisms, with maximal efficacy at 300 mg/kg. However, it failed to prevent early proximal tubular injury as reflected by persistent *KIM-1* elevation. These results position *BM* as a promising adjunctive agent in cisplatin-based chemotherapy regimens and justify further mechanistic and clinical translational studies.

## INTRODUCTION

Aging populations are increasingly associated with a high incidence of cancer-related disorders and mortality worldwide [1]. Cisplatin, a platinum-based chemotherapeutic agent, is used in several malignancies, including breast, testicular, lung, brain, esophageal, and ovarian cancers [2–4]. The accumulation of reactive oxygen species (ROS) and depletion of antioxidant defenses are key factors in cell necrosis [5]. Its nonselective action poses serious risks and toxicity.

A single dose of cisplatin caused hepatotoxicity in 28% and nephrotoxicity in 36% of patients with cancer treated with cisplatin [6]. Cisplatin causes hepatotoxicity by disrupting calcium homeostasis and inducing apoptosis [7]. Cisplatin is mainly excreted by the kidneys. It accumulates in renal proximal tubular cells, forming a toxic platinum conjugate that promotes renal cell damage [8]. Cisplatin-induced acute kidney injury (AKI) is caused by oxidative stress, inflammation, mitochondrial dysfunction, DNA adducts, and direct cytotoxicity [9, 10]. There is no clinically used curative or preventive drug for cisplatin-induced nephrotoxicity.

Kidney Injury Molecule-1 (KIM-1), a transmembrane protein, becomes significantly elevated in the proximal tubular epithelial cells following nephrotoxic injury caused by cisplatin. Unlike traditional markers such as serum urea and creatinine, which typically rise only after extensive nephron damage and a decrease in glomerular filtration rate (GFR), KIM-1 acts as a highly sensitive and specific early indicator of proximal tubular injury, particularly relevant in cisplatin-induced nephrotoxicity. Its expression serves as a valuable biomarker for early AKI detection in both preclinical and clinical contexts, as it is markedly increased in the injured proximal tubular cells and appears in urine well before traditional markers like serum creatinine show changes [11].

*Bacopa monnieri* (BM), commonly known as Brahmi, has been used as a memory enhancer in Ayurvedic practice [12]. Clinical studies suggest that BM improves cognitive performance across age groups, including attention, memory, and reasoning [13, 14]. It has also been reported to improve emotional well-being and general health and to reduce anxiety, depression, and pain [15, 16]. Evidence also indicates that it reduces stress and improves mood and sleep outcomes in children with inattention and hyperactivity [17, 18]. Previous studies have linked the presence of steroidal saponins known as bacosides to the medicinal qualities of BM extracts, including anti-inflammatory, antioxidant, cardiotonic, anti-hypercholesterolemic, and nerve tonic effects [19–22]. In addition to saponins, it contains alkaloids, flavonoids, glycosides, and cucurbitacin as bioactive constituents that contribute to its medicinal value [23].

Other botanicals, including silymarin, curcumin, and *Withania somnifera*, have also shown protective effects against cisplatin-induced organ toxicity. Our study is the first to demonstrate dual hepatorenal protective effects of BM, which is significant because cisplatin affects both organs simultaneously. Moreover, our study is the first to use the early renal injury biomarker KIM-1, providing more sensitive and translationally relevant evidence than earlier reports that relied only on conventional markers.

The addition of KIM-1 to a phytotherapeutic model is a novel methodological step that supports the development of biomarker-driven herbal pharmacology. The wide range of bioactive components, including bacosides, flavonoids, phenolic acids, and polyunsaturated fatty acids, along with its well-established neuroprotective and organ-protective properties, make it a unique candidate among the majority of other herbs examined. Cisplatin has increasingly been associated with cognitive impairment, or “chemobrain.”

Emerging anticancer activities have been reported against breast, prostate, and hepatocellular carcinoma. These broader therapeutic actions provide an additional advantage of Bacopa [24, 25]. Its established human safety profile enhances its translational value in oncology settings. Collectively, these characteristics distinguish BM from other herbal remedies and demonstrate its potential as a supportive care adjunct in cisplatin therapy.

Although cisplatin remains a cornerstone chemotherapeutic agent for a broad spectrum of malignancies, its clinical use is frequently limited by severe, dose-dependent hepatorenal toxicity driven by excessive ROS generation, antioxidant depletion, inflammation, and direct cytotoxicity to hepatocytes and proximal tubular epithelial cells. Several botanicals, including silymarin, curcumin, and *W. somnifera*, have demonstrated partial protective effects against cisplatin-induced organ damage; however, evidence regarding BM in this context is extremely limited. Existing reports on BM are sparse, fragmented, and confined to either isolated renal or hepatic endpoints, without simultaneous assessment of dual hepatorenal protection. Furthermore, prior investigations have relied exclusively on late-stage conventional biomarkers such as serum creatinine, urea, aspartate aminotransferase, and alanine aminotransferase, which lack the sensitivity required to detect early proximal tubular injury. No previous study has integrated the highly specific, early urinary biomarker KIM-1 within a phytotherapeutic model of cisplatin toxicity. This represents a critical research gap, as concurrent hepatorenal injury is common in clinical practice, and the addition of KIM-1 to conventional and oxidative stress parameters would offer more sensitive, translationally relevant insights into the early protective potential of BM.

The present *in vivo* study aimed to systematically investigate the dose-dependent protective efficacy of standardized BM extract against cisplatin-induced acute hepatorenal toxicity in male Wistar rats. Specifically, the investigation evaluated three oral doses of BM (100, 200, and 300 mg/kg body weight daily for 10 consecutive days) administered concomitantly with a single intraperitoneal dose of cisplatin (7.5 mg/kg on day 7). Comprehensive assessments included serum liver and kidney function tests, tissue levels of malondialdehyde, reduced glutathione, superoxide dismutase, and catalase, urinary KIM-1 concentrations as an indicator of early proximal tubular damage, and semi-quantitative histopathological scoring of inflammatory infiltration, necrosis, hydropic degeneration, sinusoidal dilatation, and fatty change in liver and kidney tissues. By employing this multifaceted approach, the study sought to establish the optimal therapeutic dose of BM, elucidate its predominant antioxidant mechanisms, and provide robust preclinical evidence for its potential as a safe, natural adjunctive agent capable of mitigating cisplatin-associated hepatorenal toxicities in oncology settings.

## MATERIALS AND METHODS

### Ethical approval

The present *in vivo* study involving experimental animals was reviewed and approved by the Institutional Animal Ethics Committee (IAEC) of Kasturba Medical College, Manipal Academy of Higher Education, Manipal, Karnataka, India (approval reference number: IAEC/KMC/46/2023, dated [6 May 2023]). The experimental protocol was designed and conducted in strict accordance with the guidelines and principles outlined in the Committee for Control and Supervision of Experiments on Animals (CCSEA), Government of India, Ministry of Fisheries, Animal Husbandry and Dairying (as per the Breeding of and Experiments on Animals Control and Supervision) Rules, 1998, and subsequent amendments.

All procedures adhered to the fundamental principles of the 3Rs (Replacement, Reduction, and Refinement). Replacement was addressed by selecting an established rodent model that is widely accepted for preclinical evaluation of cisplatin-induced hepatorenal toxicity and phytotherapeutic interventions. Reduction was achieved by determining the minimal sample size (n = 6 per group) through power analysis based on preliminary data and previously published studies reporting effect sizes for similar biochemical and histopathological endpoints, ensuring sufficient statistical power (≥ 80%) to detect meaningful differences while minimizing animal numbers. Refinement measures included the use of appropriate anesthesia (ketamine 80–100 mg/kg and xylazine 5–10 mg/kg intraperitoneally) for terminal procedures, provision of environmental enrichment (polypropylene cages with rice husk bedding), maintenance under controlled conditions (12:12 h light/dark cycle, 28°C ± 2°C temperature, 50 ± 10% relative humidity), and ad libitum access to standard rodent chow and filtered water.

Humane endpoints were predefined in accordance with CCSEA and institutional ethical standards. Animals were monitored daily throughout the experimental period for clinical signs of distress, including but not limited to: body weight loss exceeding 20% of initial weight, persistent lethargy, abnormal posture, hunched appearance, reduced food and water intake, piloerection, porphyrin staining, or any overt signs of severe morbidity or pain. Any animal reaching these predefined humane endpoints was immediately and humanely euthanized using an overdose of anesthetic agents followed by cervical dislocation. No animal in the present study met the criteria for premature euthanasia.

The study was reported in compliance with the ARRIVE (Animal Research: Reporting of *in vivo* Experiments) 2.0 guidelines to ensure transparency, reproducibility, and scientific rigor. All personnel involved in animal handling, dosing, sample collection, and euthanasia were adequately trained and certified in laboratory animal science and ethical practices as per institutional and national requirements.

Thirty male Albino Wistar rats, weighing between 150 and 250 g and aged 6-8 weeks, were obtained from the “Central Animal Research Facility.” The animals were housed in polypropylene cages with rice husk bedding. They were kept on a 12:12 h light/dark cycle at 28°C and 50% humidity, with free access to food and water.

### Study period and location

The research was carried out between December 2023 and February 2024 at Kasturba Medical College in Manipal, India.

### Chemicals and reagents used

Cisplatin injection was obtained from GLS Pharma Limited, Hyderabad, India (Lot No. CSIB2212G). The chemicals 2-thiobarbituric acid (TBA), trichloroacetic acid (TCA), 5,5’-dithiobis 2-nitrobenzoic acid (DTNB), 3% hydrogen peroxide, nitro blue tetrazolium, sodium carbonate, hydroxylamine hydrochloride, sodium chloride, potassium chloride, sodium phosphate dibasic, potassium phosphate monobasic, and sodium hydroxide were sourced from Sigma Aldrich, St. Louis, MO, USA, with respective lot numbers: BCCN3763, SDBB4937, STBG6026V, STBL4103, 0000469877, 0000498248, MKCP8125, SDBB6562, 0000498824, 0000468889, MKCZ6855, and 0000511658. Kits for evaluating aspartate aminotransferase (AST), alanine aminotransferase (ALT), alkaline phosphatase (ALP), total protein, and albumin were purchased from Aspen Laboratories Pvt. Ltd, Delhi, India, with catalog numbers AST-L-1149, ALT-1179, ALP-L-1143, TPR-L-068, and ALB-L-080. Urea and creatinine diagnostic kits (Catalog Nos. 1102250075 and 1102080040) were bought from Coral’s Laboratory in Uttarakhand, India. The rat Kim-1 ELISA kit (Elabscience, Texas, USA; Catalog No. E-EL-R3019) was used following the manufacturer’s instructions. Bicinchoninic acid disodium salt hydrate (CAS No. 979-88-4; SKU: D8284-10G) from Sigma Aldrich, and Bovine Serum Albumin, fraction V (pH 7, 98%) (CAS No. 9048-46-8, Catalog No. 83803) from Sisco Research Laboratories Pvt. Ltd.

### Preparation and standardization of the BM extract

In this study, the BM extract (Batch No: RD/23387), commercially titled Bacomind®, was provided as a gift sample by Natural Remedies Pvt. Ltd., Bengaluru, India. A voucher specimen (No: NR002) of BM was also deposited in the herbarium at Natural Remedies Private Limited, Bangalore, India.

The extract was a high-purity ethanolic preparation derived solely from the aerial parts of BM, with no excipients added [supplementary material]. It met pharmacopoeia standards set forth in the United States Pharmacopoeia monograph for powdered Bacopa extract, assessing identity, purity, microbial load, and heavy metal limits (e.g., lead <1.0 ppm, arsenic <1.0 ppm). The extract contains 27.2% total bacosides, measured by high-performance liquid chromatography in accordance with the same monograph, which specifies a range of 20%–33%.

For the experimental work, a fresh, homogeneous suspension of the dried extract powder was prepared daily using sterile normal saline as the vehicle. Rat doses were calculated based on body weight and given orally via gavage at 100, 200, and 300 mg/kg. The suspension was well mixed before each use to ensure even dispersion and precise dosing, maintaining consistency and stability throughout the study.

### Phytochemical screening by gas chromatography–mass spectrometry (GC–MS)

The sample was extracted with ethanol and analyzed by GC–MS Shimadzu (China), specifically the GC/MS QP2020 NX model from Shimadzu Corp., Tokyo, Japan. A capillary column SH-I-5Sil MS (30 m x 0.25 mm x 0.25 µm) was used for the chromatography. The injector was set to 280°C in split mode with a split ratio of 30, and the column flow was 1.20 mL/min using helium, with a total flow of 40.2 mL/min. The oven temperature program started at 50°C for 2 min, then increased to 300°C at 15°C/min, held for 5 min, with a total run time of 23 min.

### Experimental design and treatment regimen

**Figure 1:**
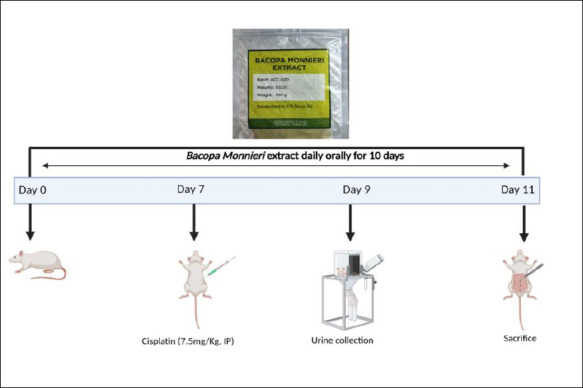
Schematic diagram of the study design.

Schematic diagram of study design is presented in Figure 1. Animals were randomly divided into five groups with six rats each. Each rat received a unique ID number (1–30) created by a computer. Randomization was done in Microsoft Excel, Office 365 (Microsoft Office, Washington, USA) using the RAND() function, giving every rat an equal chance of being placed in any of the five groups (control, cisplatin, and three BM dose groups; n = 6 each). Since the experiment took place over several days, animals were assigned in sequence following the pre-generated randomization list.

Blinding was maintained throughout histological and biochemical assessments. All samples were anonymized before analysis, and the evaluator conducting the histopathological and biochemical tests was blind to the treatment group assignments. This approach ensured an objective and unbiased evaluation of the outcomes.

Group 1 received oral normal saline daily; Group 2 received oral normal saline plus cisplatin; Group 3 received BM at 100 mg/kg daily plus cisplatin; Group 4 received BM at 200 mg/kg daily plus cisplatin; and Group 5 received BM at 300 mg/kg daily plus cisplatin. All groups were treated for 10 days. Cisplatin was given as a single intraperitoneal dose of 7.5 mg/kg on day 7. BM was administered orally 2 hours before cisplatin. Doses for BM were chosen based on prior studies [26–28]. In acute toxicity tests, a single 5000 mg/kg dose of ethanolic extract from BM’s aerial parts showed no toxicity, and in chronic toxicity tests, doses of 30, 60, 300, and 1500 mg/kg over 270 days also caused no adverse effects in rats [29]. The protective effects of BM, attributed to its antioxidant properties at doses of 100–300 mg/kg, have been reported [27, 28].

### Sample collection and tissue preparation

Animals were weighed and sacrificed on day 11 after an overnight fast, using Ketamine and Xylazine as anesthetics. Blood was drawn from the retroorbital plexus for biochemical analysis. The liver and kidneys were dissected and washed with cold saline.

Tissue homogenate preparation volumes: Tissue samples were transported and processed on ice. The excised tissue samples were weighed and homogenized in ice-cold phosphate-buffered saline (PBS, 50 mM, pH 7.4) with a 1:2 w/v ratio (1 g tissue:2.0 mL buffer). A motorized REMI homogenizer (REMI Sales & Engineering Ltd, India) was used for homogenization (10–15 strokes). Homogenates were centrifuged at 12,000 × *g* for 15 min at 4°C, and supernatants were stored at –80°C for further oxidative stress parameters analysis. Tissues were stored in 10% formalin for histopathology examination.

### Biochemical estimations

The assays were conducted in duplicate.

#### Liver function tests

AST and ALT levels were estimated using the modified International Federation of Clinical Chemistry and Laboratory Medicine method, and ALP was measured using the modified AMP (2-Amino-2-Methyl-1-propanol) method, as illustrated by Thomas [30], Moss and Henderson [31], and Tietz *et al*. [32], respectively. Total protein was measured using the Biuret method, and albumin was measured using the BCG method, as per Johnson *et al*. [33].

#### Kidney function tests

Urea and creatinine levels were measured using the urease method and the modified Jaffe’s method, respectively, as described by Oltner and Sjaunja [34] and Husdan and Rapoport [35].

#### KIM-1 protein level estimation

Urine was collected from the metabolic cage on day 9 (within 48 h of the cisplatin challenge). The urine sample was centrifuged at 10,000 rpm for 5 min at –4°C. The supernatant was collected and stored at –80°C for further analysis. The assay was based on the sandwich ELISA principle [36].

#### Biomarkers of oxidative stress

Protein Normalization: Protein concentration in homogenates was measured using the bicinchoninic acid (BCA) method (a colorimetric assay that uses proteins to reduce copper (II) to copper (I), which then forms a purple-colored complex with BCA), and oxidative stress markers were expressed as milligrams of protein. Assays were performed in duplicate to ensure precision and to account for random errors.

Malondialdehyde (MDA), glutathione (GSH), catalase (CAT), and superoxide dismutase (SOD) levels were assessed using the methods described by Nourooz *et al*. [37], Ellman [38], Nabavi *et al*. [39], and Adelakun *et al*. [40], respectively.

### Histopathological investigations

Kidney and liver tissues were examined histologically using paraffin-embedded samples. Six serial sections, spaced 10 apart, were taken from each tissue block for analysis, ensuring that the same tissue area was not sampled twice. From each group, at least 10–18 s were selected for quantification. To prevent bias, slides with these sections were coded before analysis. They were then stained with hematoxylin and eosin. The slides were incubated, cooled, and immersed in xylene for 30 min to remove the wax, then hydrated through an alcohol series of 50%, 70%, and 90%. Hematoxylin staining was followed by washing in distilled water and tap water to a bluish color. Eosin staining was then performed. After dehydration, the sections were kept in xylene for 5–10 min and mounted with Dibutylphthalate Polystyrene Xylene under a cover slip. The stained sections were examined under a light microscope (Magnus, Olympus) at 40X magnification to identify inflammatory cell infiltration, fatty liver, dilated hepatic sinusoids, and hydropic degeneration in the liver, as well as inflammatory infiltration and tubular epithelial necrosis in the kidney. Lesions were graded according to the number of affected areas, as described by Ekinci *et al*. [41] and Ishak *et al*. [42].

### Statistical analysis

Values were presented as mean ± SD or median (Q1–Q3), depending on the data. All statistical analyses were conducted using SPSS software (version 30.0, IBM Corp., NY, USA) and GraphPad Prism (Version 8.4.3, GraphPad Software, Inc., CA, USA). For variables that did not meet normality assumptions (Shapiro–Wilk test) or homogeneity of variances (Levene’s test), group comparisons were performed with the Kruskal–Wallis test, followed by pairwise Mann–Whitney U tests. Data meeting these assumptions were analyzed using analysis of variance with Tukey’s post hoc test. Exact p-values are reported with Bonferroni correction for multiple comparisons (significance threshold p < 0.005 for 10 tests across five groups). For within-group comparisons, a paired t-test was used. A p-value ≤ 0.05 was considered statistically significant.

Supplementary Table 2 displays the effect sizes and measures of precision. For variables that satisfied parametric assumptions in sensitivity analyses, we reported mean differences (relative to cisplatin) along with 95% confidence intervals and Cohen’s *d* as the effect size. For outcomes that did not meet parametric criteria, we reported p-values and effect sizes based on the rank-based U statistic (r = 1 – 2U/(n_1__2_)), where U is the Mann–Whitney U statistic, n_1_ is the sample size of group 1, and n_2_ is the sample size of Group 2. This approach ensures transparent and consistent reporting of the magnitude of differences between groups.

**Table 1:** Liver and kidney function tests among the different groups.

Parameter	Control	Cisplatin	Cis + BM100	Cis + BM200	Cis + BM300
Liver					
ALP (U/L)	346.5 (39.7)	763.5 (39.4)^a^	706.1 (16.2)^a^	589.7 (51.9)^abc^	469.7 (37.4)^abcd^
AST (U/L)	95.3 (10.9)	173.4 (5.4)^a^	161.8 (7.5)^a^	132.9 (11.9)^abc^	103.6 (6.1)^bcd^
ALT (U/L)	36.2 (4.8)	73.2 (4.6)^a^	66.9 (5.8)^a^	52.3 (8.2)^abc^	40.2 (3.9)^bcd^
Total protein (g/dL)	6.6 (6.28–6.63)	3.81 (2.95–4.69)^a^	4.71 (4.36–4.88)^a^	5.11 (4.91–5.77)^a^	6.11 (5.48–6.44)^bc^
Albumin (g/dL)	3.3 (0.35)	1.6 (0.28)^a^	1.88 (0.24)^a^	2.1 (0.41)^a^	2.7 (0.37)^abcd^
Kidney					
Urea (mg/dL)	41.2 (37.8–42.8)	59.7 (56.2–64)^a^	54.4 (53.3–57.5)^a^	51.8 (48–57.4)^a^	47.61 (46–49.52)^abc^
Creatinine (mg/dL)	0.45 (0.1)	1.5 (0.1)^a^	1.4 (0.1)^a^	1.2 (0.13)^abc^	0.82 (0.12)^abcd^

Data are presented as mean ± SD (n = 6/group) for normally distributed variables and as median (Q1–Q3) for non-normally distributed variables. Intergroup comparisons were performed using one-way analysis of variance followed by Tukey’s post hoc test for parametric data and the Kruskal–Wallis test followed by pairwise Mann–Whitney U tests for nonparametric data. For five groups (10 pairwise comparisons), p-values were adjusted using the Bonferroni correction (α = 0.05/10 = 0.005). a p < 0.05 compared to control, b p < 0.05 compared to cisplatin group, c p < 0.05 compared to Cis + BM100 group, d p < 0.05 compared to Cis + BM200 group. ALP = Alkaline phosphatase, ALT = Alanine aminotransferase, AST = Aspartate aminotransferase, Cis + BM = Cisplatin with *Bacopa monnieri*, SD = Standard deviation.

**Table 2:** Oxidative stress markers in liver and kidney tissues among the different groups.

Parameter	Control	Cisplatin	Cis + BM100	Cis + BM200	Cis + BM300
Liver					
MDA (mM/mg protein)	0.42 (0.41–0.45)	1.23 (1.2–1.3)^a^	1.2 (1.2–1.2)^a^	1.13 (1–1.2)^abc^	0.81 (0.8–0.9)^abcd^
GSH (mM/mg protein)	134.14 (132.1–137.1)	112 (100–114.3)^a^	117.1 (115.7–119.1)^ab^	121 (120.4–123.3)^ab^	125.9 (124.9–126.9)^abcd^
SOD (U/mg protein)	2.5 (2.3–2.6)	1.4 (1.3–1.4)^a^	1.5 (1.5–1.6)^ab^	1.61 (1.58–1.61)^ab^	1.67 (1.65–1.69)^abcd^
CAT (U/mg protein)	31.3 (29.8–32.4)	13 (12.6–13.6)^a^	16.6 (15.4–17.11)^ab^	18 (17.8–18.2)^abc^	18.7 (18.4–19.1)^abcd^
Kidney					
MDA (mM/mg protein)	0.47 (0.01)	1.3 (0.04)^a^	1.2 (0.03)^a^	1.15 (0.1)^ab^	0.91 (0.1)^abcd^
GSH (mM/mg protein)	131.8 (131.1–133.4)	107.4 (94.5–112)^a^	115.4 (111.2–117.4)^a^	118.5 (116.14–121.3)^ab^	124.3 (119–125.9)^abc^
SOD (U/mg protein)	2.27 (2.19–2.36)	1.24 (1.22–1.26)^a^	1.3 (1.28–1.32)^a^	1.4 (1.3–1.4)^ab^	1.42 (1.38–1.46)^abc^
CAT (U/mg protein)	32.6 (0.65)	15.5 (0.64)^a^	17.01 (0.42)^ab^	18.1 (0.17)^abc^	18.9 (0.33)^abcd^

Data are presented as mean ± SD (n = 6/group) for normally distributed variables and as median (Q1–Q3) for non-normally distributed variables. Intergroup comparisons were performed using one-way analysis of variance followed by Tukey’s post hoc test for parametric data and the Kruskal–Wallis test followed by pairwise Mann–Whitney U tests for nonparametric data. For five groups (10 pairwise comparisons), p-values were adjusted using the Bonferroni correction (α = 0.05/10 = 0.005). MDA = Malondialdehyde, GSH = Reduced glutathione, SOD = Superoxide dismutase, CAT = Catalase, Cis + BM = Cisplatin with *Bacopa monnieri*.

a p < 0.05 compared to control group,

^b^ p < 0.05 compared to cisplatin group, ^c^ p < 0.05 compared to Cis + BM100 group, ^d^ p < 0.05 compared to Cis + BM200 group.

## RESULTS

### Phytochemical screening

A total of 76 compounds were identified, with auto-integration performed on the top 29 peaks, sorted by height. The screening revealed a diverse range of chemical classes in the extract, including phenolic acid (benzoic acid), fatty acids (n-hexadecanoic acid), fatty acid esters (9-octadecenoic acid, d-mannitol, hexadecanoic acid), aromatic hydrocarbons (1,2-diphenylcyclopropane, cyclohexane), diterpenes (neophytadiene), polyunsaturated fatty acids (9,12-octadecadienoic acid), fatty aldehydes (9-octadecenal, tricosanal), phosphine oxides (triphenylphosphine oxide), phenolic glycosides (salicin), phytosterols (stigmasterol), and phenols (2-methoxy-4-vinylphenol, 4-vinylbenzene-1,2-diol). The data for the compounds with the highest peak intensity in the ethanolic extract of BM are included in Supplemental Table 1.

**Figure 2:**
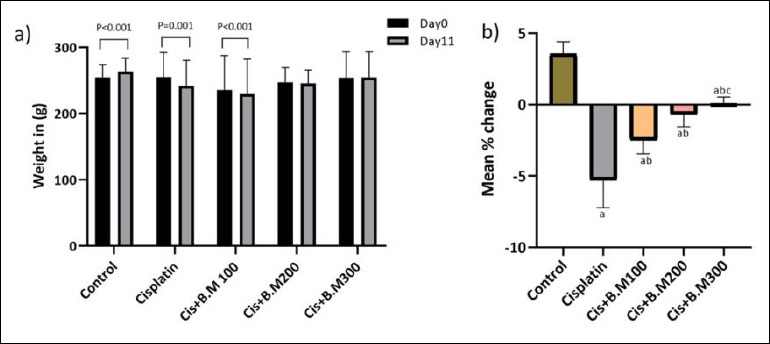
Effects of treatment on body weight across the experimental groups. (a) Changes in body weight over the experimental period and (b) Mean percentage change in body weight among groups. Data are expressed as mean ± standard deviation (n = 6/group). Cis + BM = Cisplatin with *Bacopa monnieri*.^a^ p < 0.05 compared to control group, ^b^ p < 0.05 compared to cisplatin group, ^c^ p < 0.05 compared to Cis + BM100 group. Statistical analyses were performed using paired t-tests for within-group comparisons over time and one-way analysis of variance followed by Tukey’s post hoc test for intergroup multiple comparisons.

Body weight increased significantly in the control group (p < 0.001) but decreased in the cisplatin (p = 0.001) and BM100 groups (p < 0.001) compared to baseline values. The weights of animals in the BM200 and BM300 groups showed no significant difference (Figure 2a).

Figure 2b shows the mean percentage change in body weight from baseline to day 11. Animals in the control and Cis + BM300 groups gained weight, whereas those in the cisplatin and Bacopa groups lost weight. Weight loss was more pronounced in the cisplatin group. Compared with the control group, weight loss was significantly greater in all the groups receiving cisplatin (p < 0.001), Cis + BM100 (p < 0.001), Cis + BM200 (p < 0.001), and Cis + BM300 (p < 0.001). Weight loss was less in the Cis + BM100 (p = 0.002) and Cis + BM200 (p < 0.001) groups than in the cisplatin group. Among the Bacopa groups, Cis + BM300 showed an improvement in weight compared with the Cis group (p < 0.001) and the Cis + BM100 group (p = 0.005).

### Biochemical estimations

As shown in Table 1, levels of ALP, AST, and ALT were significantly higher in all groups compared to the control. However, in the Cis + BM300 group, the increases in AST and ALT were not statistically significant. Both the Cis + BM200 and Cis + BM300 groups exhibited reductions in ALP, AST, and ALT levels relative to the cisplatin group, with all three enzymes showing highly significant differences (p < 0.001). Among the Bacopa-treated groups, the Cis + BM 300 group had lower levels of ALP, AST, and ALT than the Cis + BM 100 group, with all comparisons reaching high statistical significance (p < 0.001). Compared to the Cis + BM200 group, the Cis + BM300 group showed significantly lower levels of ALP (p < 0.001), AST (p < 0.001), and ALT (p = 0.009). Additionally, Cis + BM200 significantly reduced the levels of all three enzymes compared to Cis + BM100 (all p ≤ 0.001). The total protein concentration was significantly decreased in the cisplatin, Cis + BM100, and Cis + BM200 groups compared to the control group (all p = 0.004). In contrast, the Cis + BM300 group maintained total protein levels, showing no significant difference from the control. The albumin concentration was markedly reduced in the cisplatin group (p < 0.001), Cis + BM100 (p < 0.001), Cis + BM200 (p < 0.001), and Cis + BM300 (p = 0.015) compared to the control. Compared to the cisplatin group, Cis + BM300 significantly increased total protein (p = 0.004) and albumin (p < 0.001) levels. Among the Bacopa-treated groups, the Cis + BM300 group showed a significant increase in total protein (p = 0.004) and albumin levels (p = 0.003) compared to the Cis + BM100 group. Albumin levels also rose in the Cis + BM 300 group (p = 0.048) compared with the Cis + BM 200 group.

Urea levels were significantly elevated in all groups compared with the control group. Compared with the cisplatin group, urea levels were significantly lower in the Cis + BM300 group (p = 0.004). Among the Bacopa-treated groups, urea levels were significantly lower in Cis + BM300 (p = 0.004) than in Cis + BM100.

Creatinine levels were significantly higher in all groups than in the control group. Compared with the cisplatin group, creatinine levels were significantly lower in the Cis + BM200 (p < 0.001) and Cis + BM300 (p < 0.001) groups. Among the Bacopa-treated groups, creatinine levels were significantly lower in the Cis + BM200 (p=0.003) and Cis + BM300 (p < 0.001) groups than in the Cis + BM100 group. A further significant reduction was observed in the Cis + BM300 group compared with the Cis + BM200 group (p < 0.001).

### Effect on the KIM-1 protein levels

**Figure 3:**
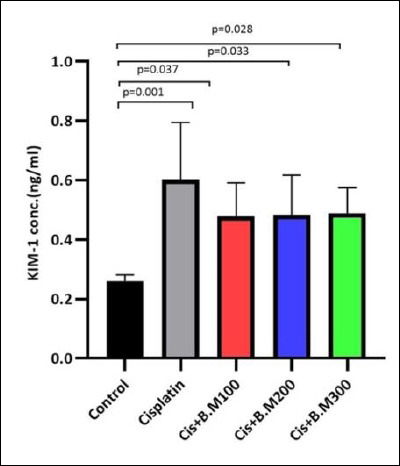
Kidney injury molecule-1 (KIM-1) protein levels in urine across the different groups. Data are expressed as mean ± standard deviation (n = 6 per group). Statistical significance was assessed using one-way analysis of variance followed by Tukey’s post hoc test. Cis + BM = Cisplatin with *Bacopa monnieri*.

KIM-1 levels showed a significant increase in the cisplatin group (p = 0.001), as well as in the Cis + BM100 (p = 0.037), Cis + BM200 (p = 0.033), and Cis + BM300 (p = 0.028) groups. However, there were no significant differences between the cisplatin group and the Bacopa-treated groups (Cis + BM100, Cis + BM200, Cis + BM300) or among the Bacopa-treated groups themselves (Figure 3).

### Biomarkers of oxidative stress

The hepatic levels of GSH, SOD, and CAT were significantly lower in all treatment groups than in the control (Table 2). When compared to the cisplatin group, all Bacopa-treated groups, Cis + BM100, Cis + BM200, and Cis + BM300, exhibited significant rises in GSH, SOD, and CAT levels (p = 0.004 for all comparisons).

The Cis + BM300 group among the Bacopa-treated groups showed a significantly greater increase in GSH, SOD, and CAT levels compared to the Cis + BM100 group (all p = 0.004). Specifically, CAT levels were notably higher in the Cis + BM300 group (p = 0.004) than in the Cis + BM200 group, and in the Cis + BM200 group (p = 0.004) compared to the Cis + BM100 group, indicating a dose-dependent response. Only CAT levels were significantly elevated in the Cis + BM200 group (p = 0.004) versus Cis + BM100. GSH, SOD, and CAT levels were higher in the Cis + BM300 group than in the Cis + BM200 group. MDA levels were increased in all groups relative to the control. Compared to the cisplatin group, the Cis + BM100 group showed no significant change, but MDA levels were significantly lower in the Cis + BM200 (p = 0.004) and Cis + BM300 (p = 0.004) groups. Within the Bacopa-treated groups, the Cis + BM300 dose yielded the lowest MDA levels, with significant reductions compared to BM100 (p = 0.004) and BM200 (p = 0.004). Additionally, MDA levels in the Cis + BM200 group were lower than in the Cis + BM100 group (p = 0.004).

In renal tissue, GSH, SOD, and CAT levels were significantly decreased in all groups compared with the control group. Compared with the cisplatin group, treatment with Bacopa at 200 and 300 mg/kg significantly restored GSH (p = 0.004), SOD (p = 0.004), and CAT (p < 0.001). Only CAT levels were significantly elevated in the Cis + BM100 group compared with the cisplatin group (p < 0.001).

Among the Bacopa-treated groups, the Cis + BM300 group showed significantly higher levels of GSH (p = 0.004), SOD (p = 0.004), and CAT (p < 0.001) compared to the Cis + BM100 group. Additionally, CAT levels were notably higher in the Cis + BM300 group (p = 0.036) than in the Cis + BM200 group, and higher in the Cis + BM200 group (p = 0.007) than in the Cis + BM100 group, indicating a dose-dependent increase.

MDA levels were substantially higher in all experimental groups compared to the control (p < 0.001). When compared to the cisplatin group alone, MDA levels decreased in the Cis + BM200 (p = 0.010) and Cis + BM300 (p < 0.001) groups. Among the groups treated with Bacopa, MDA levels were notably lower in the Cis + BM300 group than in the Cis + BM100 and Cis + BM200 groups, both with p < 0.001. There was no significant difference between the Cis + BM100 and Cis + BM200 groups.

### Effect on the liver histopathology

**Figure 4:**
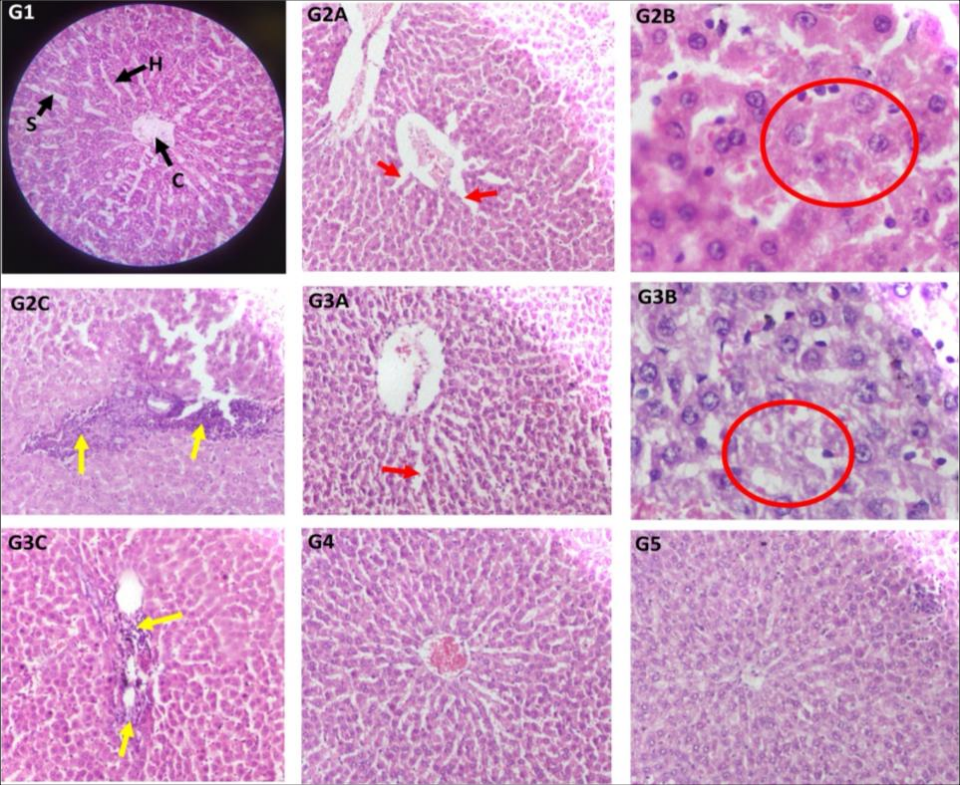
Representative photomicrographs of liver sections stained with haematoxylin and eosin. Groups: Control (G1); cisplatin (G2A, G2B, G2C); Cis + BM100 (G3A, G3B, G3C); Cis + BM200 (G4); Cis + BM300 (G5), observed under a digital microscope. Magnification: 40× objective × 10× eyepiece and 10× objective × 10× eyepiece. Key structures: central vein (C); hepatic cords (H); hepatic sinusoids (S). Pathological findings: (G2A, G3A) Mild dilatation of hepatic sinusoids, indicated by red arrows. (G2B, G3B) Hydropic degeneration of hepatocytes, outlined by red circles. (G2C, G3C) Inflammatory cell infiltration, marked by yellow arrows. Cis + BM = Cisplatin with *Bacopa monnieri*.

Using a digital microscope, the main components of the liver, such as the hepatocellular architecture and biliary system, were examined (Figure 4). The control group displayed typical liver structure, including hexagonal

hepatic lobules, a central vein, and a normal arrangement of hepatic cords radiating from the central vein. The portal triads, consisting of the biliary ductule, portal venule, and hepatic arteriole, were also properly located and patterned. The cisplatin group exhibited abnormal hepatic cords; hepatocytes showed hydropic degeneration with vacuolated cytoplasm, along with mild dilatation of hepatic sinusoids, signs of fatty liver, and infiltration of inflammatory cells in some areas. The Cis + BM 100 group showed changes similar to those seen in the Cis group. In the Cis + BM 200 group, the central vein, hepatic cords, and sinusoids appeared relatively improved compared to the control. The Cis + BM 300 group’s liver structure resembled that of the control, appearing normal. Changes in liver histopathology were scored and summarized in Table 3 based on qualitative observation [41, 42].

**Table 3:** Microscopic changes in the liver and kidney among the different groups.

Microscopic changes	Control	Cisplatin	Cis + BM100	Cis + BM200	Cis + BM300
Liver					
Inflammatory cell infiltration	0	2	1	0	0
Fatty liver	0	1	1	0	0
Dilatation of the hepatic sinusoids	0	2	1	0	0
Hydropic degeneration	0	1	1	0	0
Kidney					
Inflammatory cell infiltration	0	2	2	1	0
Tubular epithelial necrosis	0	3	2	1	0

The semi-quantitative grading was adapted from Ekinci *et al.* [41]. Each lesion was examined in six non-overlapping fields (40× magnification), and a blinded observer assigned grades. Inflammatory cell infiltration was graded by the number of affected areas per field as follows: absent (0) = ≤3; mild (1) = 4–8; moderate (2) = 9–12; severe (3) = 13–18. Other lesions (fatty change, sinusoidal dilatation, hydropic degeneration, and tubular necrosis) were scored as follows: 0 = none; mild (1) = 1–2 areas; moderate (2) = 3–4 areas; severe (3) = 5–6 areas. Cis + BM = Cisplatin with *Bacopa monnieri*.

### Effect on the histopathology of the kidneys

**Figure 5:**
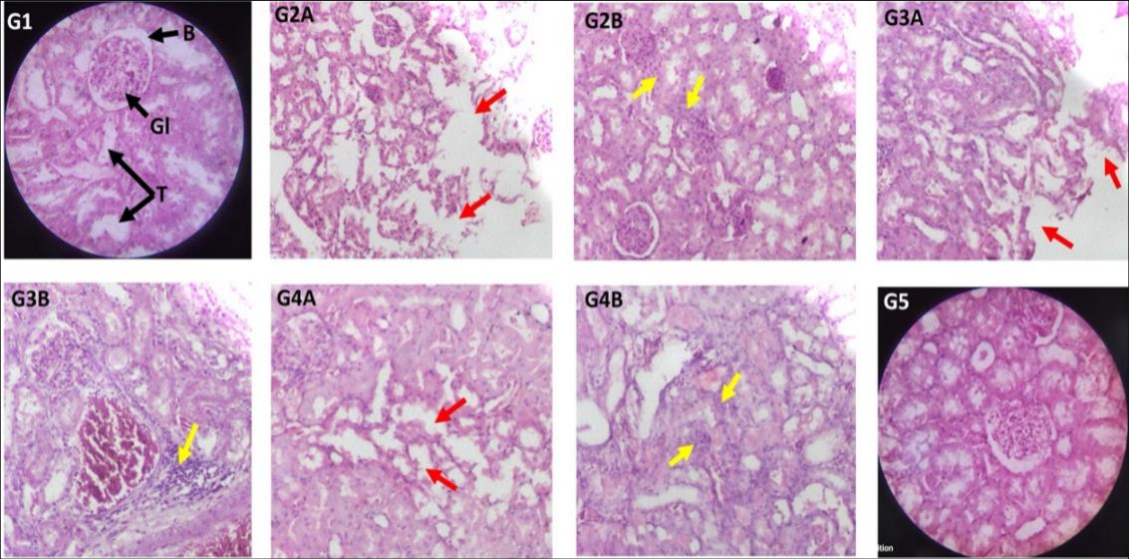
Representative photomicrographs of kidney sections stained with haematoxylin and eosin. Groups: Control (G1); cisplatin (G2A, G2B); Cis + BM100 (G3A, G3B); Cis + BM200 (G4A, G4B); Cis + BM300 (G5), observed under a digital microscope. Magnification: 40× objective × 10× eyepiece and 10× objective × 10× eyepiece. Key structures: glomerulus (G); Bowman’s capsule (B); renal tubules (T). Pathological findings: Tubular epithelial necrosis, indicated by red arrows, observed in (G2A, G3A, G4A). Inflammatory cell infiltration into the peritubular interstitial spaces, marked by yellow arrows, observed in (G2B, G3B, G4B) and additional associated fields. Cis + BM = Cisplatin with *Bacopa monnieri*. The method used to grade histopathological damage was a semi-quantitative assessment in which features such as inflammatory cell infiltrates, fatty change, sinusoidal dilatation, hydropic degeneration, and tubular necrosis were graded based on the number of areas in which they were observed [41, 42].

Sections of the renal cortex in rats from the control group displayed normal glomeruli, Bowman’s capsules, and proximal, distal, and collecting tubules (Figure 5). Groups 2 and 3 showed areas of diffuse inflammatory cell infiltration in the peritubular interstitial spaces, suggesting interstitial nephritis. These groups also exhibited severe necrosis of epithelial cells in the proximal and distal convoluted tubules. Group 4 had mild inflammatory infiltration and tubular epithelial necrosis, while Group 5 maintained normal kidney structure similar to the control. Table 3 presents the qualitative scoring of histopathological changes in the renal cortex [41, 42].

## DISCUSSION

This study aimed to explore the potential benefits of BM in cisplatin-induced hepatic and renal toxicity. Elevated liver enzyme levels are indicators of liver dysfunction [43]. Our findings showed a significant increase in serum enzyme levels after cisplatin treatment, corroborating previous research on its harmful effects on hepatocytes [44, 45]. Consistent with existing studies, cisplatin also caused notable rises in serum urea and creatinine concentrations [46]. The toxic impact of cisplatin on the liver and kidneys was mainly due to increased ROS production and reduced endogenous antioxidant defenses. This imbalance leads to cellular damage, inflammation, and apoptosis, impairing the structure and function of hepatocytes and renal tubular cells [47–50]. Conversely, BM treatment showed a dose-dependent improvement in hepatic enzyme levels and renal function markers, with complete enzyme normalization at 300 mg/kg. These findings align with earlier reports confirming BM’s effectiveness in restoring hepatic and renal functions affected by cisplatin [29, 51].

Our GC–MS analysis of BM identified several bioactive compounds known for their cytoprotective, anti-inflammatory, and antioxidant properties, likely contributing to its protection against cisplatin-induced hepatorenal oxidative damage. The extract’s saponin fraction, which includes betulinic acid, bacosides, bacopasides, and bacosaponins, is especially notable [52]. Bacosides comprise about 30% of the extract and are the primary saponins detected. Two main bacosides are Bacoside-A (B-A) and Bacoside-B, with B-A exhibiting superior pharmacological effects. Evidence highlights Bacoside-A’s role in reducing liver damage, partly due to its hydroxyl group in the phenolic ring that affords free radical scavenging activity. Administered alone, Bacoside-A restores liver enzyme levels in experimental models, underscoring its therapeutic potential [53]. Previous studies have shown the hepatoprotective effects of BM extract through antioxidant action, membrane preservation, and hepatocyte regeneration [54]. Our findings support these reports, demonstrating an increase in antioxidant defenses following treatment with BM.

Linoleic acid reduces MDA levels in LPS-induced liver damage by activating the Nrf2-NQO1 pathway, suppressing pro-inflammatory cytokines (TNF-α, IL-6), and boosting antioxidants like SOD, CAT, and GSH [55]. Additionally, 9-octadecenoic acid esters have strong anti-inflammatory and antioxidant effects, lowering ROS and NF-κB activation [56]. Stigmasterol, a phytosterol, shows hepatoprotective effects by modulating the NF-κB and Nrf2 pathways [57]. 4-Hydroxybenzoic acid scavenges ROS and enhances endogenous antioxidants via Nrf2 signaling [58]. 2-methoxy-4-vinylphenol inhibits lipid peroxidation and iNOS through HO-1 induction [59, 60]. Salicin, a phenolic glycoside, possesses antioxidant properties [61]. Consistent with our biochemical observations, sinapyl alcohol, a phenylpropanoid in our extract, exhibits strong antioxidant activity by increasing SOD, CAT, and GSH levels [62], while neophytadiene, a diterpenoid hydrocarbon, reduces oxidative and inflammatory injury by suppressing NF-κB and COX-2 signaling [63]. Similar to our results of lowered oxidative and inflammatory markers, n-hexadecanoic acid inhibits inflammation by blocking phospholipase A_2_ a key enzyme in arachidonic acid metabolism [64], and adenosine, a cytoprotective modulator that preconditions renal and hepatic cells via A-A receptor pathways, reducing oxidative stress and preserving mitochondrial function [65, 66]. Overall, these combined mechanisms of Bacopa-derived compounds explain the significant decrease in lipid peroxidation (MDA), increased enzymatic antioxidants (GSH, SOD, CAT), and the normalization of liver and kidney function markers, confirming Bacopa’s protective role against cisplatin toxicity through its multifaceted antioxidant and anti-inflammatory actions.

Bacopa primarily reduces pro-inflammatory mediators by inhibiting NF-κB, COX-2, iNOS, and cytokines, while activating the Nrf2 antioxidant pathway and enhancing defenses like SOD, CAT, and GSH. Some reports also indicate that B. melanogaster offers additional protection through mechanisms such as safeguarding mitochondria by preventing damage and ROS buildup, and fostering an anti-apoptotic environment marked by lower Bax and caspase-3 activity and higher Bcl-2 levels [67]. These actions demonstrate the wider therapeutic potential of BM beyond antioxidant effects, underscoring its importance in reducing cisplatin-induced damage.

Previous research has confirmed that BM offers therapeutic benefits in reducing renal failure in diabetic mouse models. This effect is linked to B-A’s ability to boost GSH levels, improve key antioxidant enzyme functions, and decrease lipid peroxidation, indicated by lower MDA levels. B-A effectively decreased serum creatinine and urea, while neutralizing free radicals. Histopathological studies further support BM’s therapeutic potential, showing its role in restoring the structural integrity of renal tissue [68]. Our findings align with these results, demonstrating that BM significantly reduces MDA levels and enhances antioxidant enzyme activity [69, 70]. The recovery of GSH and antioxidants suggests Bacoside-A may be the main factor driving hepatorenal protection. We recommend future research to isolate and directly test Bacoside-A and other bacosides.

KIM-1 is a highly sensitive, non-invasive biomarker of proximal tubular damage. Its expression significantly increases after AKI, with the extracellular domain being released into the urine. In line with previous studies, cisplatin administration in our research also led to increased KIM-1 levels [71] (Figure 3).

Although downstream markers like urea and creatinine, along with histology, showed improvements, the fact that KIM-1 levels did not significantly decrease suggests that BM might not have fully prevented the initial epithelial damage responsible for KIM-1 expression, which is an early and specific marker of proximal tubular injury. As doses increased from 100 to 300 mg/kg, a trend of decreasing urine KIM-1 levels was noted; however, the oral doses might not have been sufficient in renal tissue to offer complete tubular protection. Using purified bacoside concentrates or further fractionation and enrichment of bacoside-rich fractions could help clarify its nephroprotective potential. Future studies with longer treatment periods, higher or optimized doses, renal pharmacokinetics to ensure adequate bioavailability, and administration methods such as parenteral routes that bypass first-pass metabolism or nano-formulations will offer further insights into renal pharmacokinetics.

Our study results were confirmed by tissue histopathology. The liver and kidney tissues were examined histologically following Ekinci *et al*. [41]. Cisplatin caused severe inflammatory cell infiltration, fatty deposits, dilated hepatic sinusoids, and hydropic degeneration. Importantly, BM at doses of 200 and 300 dilutions restored liver structure and function. Similarly, in the kidneys, BM at 300 mg/kg reversed cisplatin-induced inflammatory infiltration and tubular epithelial necrosis.

### Strengths

Adding KIM-1 into a phytotherapeutic model is a new approach that supports biomarker-based herbal pharmacology, although its decrease was not statistically significant. The dose-response experiment (100, 200, and 300 mg/kg) showed a clear therapeutic window, with 300 mg/kg nearly fully restoring renal and liver functions. GC–MS profiling identified 76 compounds, offering mechanistic insight beyond crude extract studies by linking bacosides, sterols, phenolic acids, and fatty acids to functional and histological recovery. Besides its antioxidant, anti-inflammatory, and anti-apoptotic effects relevant to cisplatin-induced liver and kidney damage, BM is also known for cognition enhancement and anticancer effects against breast, prostate, and hepatocellular carcinoma. These wide-ranging therapeutic properties give Bacopa an advantage over many other herbal agents in managing cisplatin toxicity.

### Limitations

This study used a rat model to explore mechanisms, but it does not fully represent human cisplatin-induced hepatorenal toxicity. Differences in drug metabolism, immune responses, and repair mechanisms between rodents and humans may affect the level of apparent protection. Additionally, the Bacopa dose given to animals may not be directly applicable to humans, considering variations in absorption, distribution, metabolism, and elimination. Without pharmacokinetic and dose-translation research, it is challenging to predict if similar protective effects would occur in humans. The study only examined short-term results, so the potential benefits of Bacopa for long-term or chronic cisplatin-induced liver and kidney damage remain unclear. Long-term functional metrics like survival, GFR, and other physiological indicators were not assessed to verify sustained effects. Female animals were excluded, limiting insights into sex-related response differences. While antioxidant activity was identified as a key mechanism, other pathways such as apoptosis markers, cytokines, and mitochondrial gene expression should also be investigated. Despite Bacopa’s protective effects, its clinical significance is still uncertain, underscoring the need for carefully designed human trials before recommending it as an adjunct therapy for cisplatin patients.

## CONCLUSION

The present *in vivo* study demonstrated that standardized BM extract exerted significant, dose-dependent hepatorenal protection against cisplatin-induced toxicity in male Wistar rats. At 200 and 300 mg/kg, BM markedly attenuated elevations in serum alkaline phosphatase, aspartate aminotransferase, and alanine aminotransferase (all p < 0.001 versus cisplatin group), restored total protein and albumin levels toward control values at the highest dose, and significantly reduced urea (300 mg/kg) and creatinine (200 and 300 mg/kg; p < 0.001). Oxidative stress markers improved dose-dependently, with elevated glutathione, superoxide dismutase, and catalase activities and lowered malondialdehyde concentrations in both liver and kidney tissues (p < 0.001 at higher doses). These biochemical recoveries were strongly corroborated by histopathological evidence of reduced inflammatory cell infiltration, tubular epithelial necrosis, hydropic degeneration, sinusoidal dilatation, and fatty change, with near-normal tissue architecture restored at 300 mg/kg. Urinary Kidney Injury Molecule-1 (KIM-1) levels, however, remained elevated across all cisplatin-exposed groups, indicating incomplete prevention of early proximal tubular injury.

The findings position BM as a safe, cost-effective, and readily available phytotherapeutic adjunct that could be integrated into supportive care protocols during cisplatin-based chemotherapy. By mitigating hepatorenal toxicities through antioxidant mechanisms without interfering with the primary anticancer action of cisplatin, BM has the potential to improve patient tolerability, reduce dose reductions or treatment interruptions, and enhance overall quality of life in oncology settings, particularly in resource-limited healthcare environments where standardized herbal formulations are accessible.

Subsequent research should focus on bioactivity-guided fractionation to identify and isolate the principal bioactive constituents (especially bacosides), long-term chronic exposure models to evaluate sustained efficacy and safety, detailed pharmacokinetic and pharmacodynamic profiling (including renal tissue distribution), and molecular pathway analyses beyond antioxidation (e.g., Nrf2 activation, NF-κB inhibition, and anti-apoptotic signaling). Most importantly, well-designed randomized clinical trials in cancer patients receiving cisplatin are essential to confirm translational benefits, optimal dosing, and absence of herb–drug interactions.

In conclusion, BM extract emerges as a promising natural hepatoprotective and nephroprotective agent against cisplatin-induced toxicity, with maximal efficacy observed at 300 mg/kg through potent antioxidant restoration and histopathological preservation. While the lack of significant KIM-1 reduction highlights a limitation in early tubular protection, the overall preclinical evidence strongly supports further development of BM as an adjunctive strategy in modern oncology, bridging traditional Ayurvedic knowledge with contemporary cancer supportive care.

## DATA AVAILABILITY

The supplementary data can be made available from the corresponding author upon request.

## AUTHORS’ CONTRIBUTIONS

SS: Conducted the study, drafted the initial manuscript, developed the methodology, collected the data, and carried out the formal analysis. SG: Contributed to conceptualization, reviewed and edited the manuscript, wrote specific sections, curated the data, and performed the formal analysis. RM: Provided supervision, authored, reviewed, and edited the manuscript, and conducted biochemical analyses. MR: Contributed to writing, reviewing, and editing the manuscript, as well as preparing and analyzing histological reports. SK: Offered supervision and participated in writing, reviewing, and editing. SA also provided supervision and contributed to drafting and revising the manuscript. SC: Helped with the study, performed formal analysis, and assisted in editing. MB: Carried out formal analysis and contributed to writing, reviewing, and editing. All authors have read and approved the final manuscript.
